# The Role of Peroxisome Proliferator-Activated Receptors in the Esophageal, Gastric, and Colorectal Cancer

**DOI:** 10.1155/2012/242498

**Published:** 2012-09-06

**Authors:** Alessandra Fucci, Tommaso Colangelo, Carolina Votino, Massimo Pancione, Lina Sabatino, Vittorio Colantuoni

**Affiliations:** Department of Biological, Geological and Environmental Sciences, University of Sannio, 82100 Benevento, Italy

## Abstract

Tumors of the gastrointestinal tract are among the most frequent human malignancies and account for approximately 30% of cancer-related deaths worldwide. Peroxisome proliferator-activated receptors (PPARs) are ligand-activated transcription factors that control diverse cellular functions such as proliferation, differentiation, and cell death. Owing to their involvement in so many processes, they play crucial roles also in the development and physiology of the gastrointestinal tract. Consistently, PPARs deregulation has been implicated in several pathophysiological conditions, including chronic inflammation and cancer development. This paper summarizes the current knowledge on the role that the various PPAR isoforms play in the pathogenesis of the esophageal, gastric, and intestinal cancer. Elucidation of the molecular mechanisms underlying PPARs' signaling pathways will provide insights into their possible use as predictive biomarkers in the initial stages of the process. In addition, this understanding will provide the basis for new molecular targets in cancer therapy and chemoprevention.

## 1. Introduction

Peroxisome proliferator-activated receptors (PPARs) are ligand-activated transcription factors belonging to the nuclear hormone receptor superfamily. Three subtypes, PPAR*α*, PPAR*β*/*δ*, and PPAR*γ*, have been identified so far. PPAR*α* is expressed in the liver, kidney, small intestine, heart, and muscle, where it activates fatty acid catabolism and controls lipoprotein assembly in response to long-chain unsaturated fatty acids, eicosanoids, and hypolipidemic drugs (e.g., fenofibrate) [1, 2]. PPAR*β*/*δ* is more broadly expressed and is implicated in fatty acid oxidation, keratinocyte differentiation, wound healing, and macrophage response to VLDL metabolism. This isoform has been implicated in transcriptional-repression functions and has been shown to repress the activity of PPAR*α* or PPAR*γ* target genes [2–7]. PPAR*γ*1 and *γ*2 are generated from a single-gene *PPARG* by differential promoter usage and alternative splicing [8–12]. PPAR*γ*1 is expressed in colon, immune system (e.g., monocytes and macrophages), and other tissues where it participates in the modulation of inflammation, cell proliferation, and differentiation. PPAR*γ*2 contains 28 additional amino acids at the N-terminus, as compared to PPAR*γ*1, and is expressed in adipose tissue where it plays a pivotal role in adipocyte differentiation, lipid storage, and energy dissipation [12]. *PPARG3* and *PPARG4* are splicing variants of *PPARG1* mRNA and give rise to the same PPAR*γ*1 protein [8, 9, 12]. Since PPAR*γ* is also involved in glucose metabolism improving insulin sensitivity, selective ligands such as the thiazolidinediones (TZD) are used as insulin-sensitizing drugs in type 2 diabetes [2, 4, 5].

 As all nuclear receptors, PPARs share a modular structure with four distinct domains [13, 14]. The A/B domain at the N-terminus is the key determinant of isotype-selective gene function and harbors a ligand-independent transcriptional activating function (AF-1) motif. The C domain is the DNA binding domain, with the typical two-zinc-finger structure with which the receptor binds the major groove of the double helix DNA of the peroxisome proliferator response elements (PPREs). They are formed by direct repeats (DRs) of the core sequence AGG(A/T)CA, separated by one or two nucleotides (DR1 and DR2, resp.). The D domain or hinge region allows receptor dimerization and DNA binding. The E/F domain contains the ligand-binding domain (LBD), a large binding pocket in which a variety of natural and synthetic ligands, such as fatty acids, eicosanoids, linoleic acid derivatives, as well as oxidized and nitrated fatty acids, accommodate. In addition, this domain exhibits the ligand-dependent transcriptional-activating function (AF2) motif on the C-terminus helix 12 [13]. Both the D and E/F domains are required to the dimerization with the 9-cis retinoic acid receptor (RXR) with which PPARs form permissive heterodimers bound to their cognate PPREs. Several genes involved in lipid metabolism and energy homeostasis, as well as genes modulating cell proliferation, differentiation, and survival, have functional PPREs in their regulatory regions [1, 2, 13, 15].

**Figure 1:**
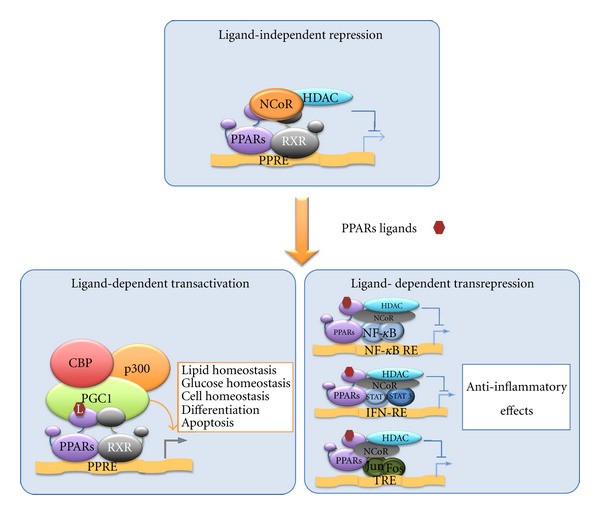
PPARs-mediated mechanisms of transcriptional regulation. In the absence of ligands, PPARs bind the promoters of their target genes and repress transcription by recruiting the corepressor complex. In the presence of ligands, PPARs can induce either ligand-dependent transactivation or transrepression. Transactivation involves PPARs heterodimerization with the retinoid X receptors (RXRs) followed by recognition of specific PPAR response elements (PPREs) and interaction with coactivators. Transrepression involves interference with other signal transduction pathways, including NF*κ*B, STAT, and AP1. NF*κ*B-RE: NF*κ*B response element; IFN-RE = “interferon-stimulated gene factor” responsive element; TRE = O-tetradecanoylphorbol 13-acetate-responsive element.

PPARs regulate gene expression through distinct mechanisms: ligand-dependent transactivation, ligand-independent repression, and ligand-dependent transrepression (Figure 1) [16, 17]. Ligand-dependent transactivation is considered the “classical mode of action” of PPARs: upon ligand binding, the helix 12 of the LBD folds back exposing the AF2 motif that governs the recruitment of transcriptional coactivators. These, in turn, facilitate the assembly of the general transcriptional machinery at PPRE-containing promoters [16, 17]. In the absence of ligand, PPARs repress transcription of target genes by recruiting corepressor complexes (e.g., NCoR and SMRT). Finally, recent studies have disclosed an additional “nongenomic” mode of action defined “transrepression” that involves gene repression in a ligand-dependent manner through protein-protein interactions with NF*κ*B, AP1, Smads, STATs, and NFATs [17–19]. In contrast to transcriptional activation and repression, transrepression does not involve binding to PPREs but is attained through the recruitment and stabilization of the corepressor complexes on the promoters of pro-inflammatory genes. This mechanism might explain the anti-inflammatory properties of PPARs [17–20]. 

## 2. Gastrointestinal Cancers 

The inner lining of the digestive tract is composed by high-proliferating cells located at the basis of the epithelium and by differentiated cells undergoing continuous replacement. The rapid cell turnover required for the maintenance of the mucosa homeostasis and the response to an adverse environment, such as toxins and carcinogens present in digested foods, makes the digestive tract a common site of cancer development in humans. In particular, esophagus, stomach, and colon are at high risk of developing cancer: indeed esophageal (EC), gastric (GC), and colorectal cancers (CRC) represent very common malignancies and account for approximately 30% of cancer-related deaths worldwide [21]. 

Esophageal cancer (EC) is the sixth most common cause of cancer-related death worldwide. Barrett's esophagus is the premalignant condition that appears to predispose to the adenocarcinoma trough a metaplasia-dysplasia-carcinoma sequence. The molecular mechanisms underlying the events leading to the conversion of the normal squamous epithelium to a metaplastic columnar epithelium are poorly understood. However, chronic activation of NF*κ*B, together with the increase of COX-2 and gastrin expression due to gastroesophageal reflux could be responsible for chronic inflammation-related cancer promotion [22, 23]. 

Gastric cancer (GC) remains the second leading cause of cancer-related death worldwide. More than 90% of these tumors are adenocarcinomas originating from the glandular epithelium of the gastric mucosa [21]. Also in this case, inflammation plays a pivotal role in tumor development. In particular, *H. pylori* infection is the major causative agent of chronic gastritis and gastrointestinal metaplasia characterized by infiltration of inflammatory cells, enhanced expression of chemokines, NF*κ*B activation, COX-2 overexpression, and upregulation of Wnt signaling pathway leading to aberrant cell proliferation, excessive angiogenesis, and inhibition of apoptosis [24]. 

Colorectal cancer (CRC) is the third most common cancer in men and the second in women [21, 25]. In the vast majority of cases, CRC occurs sporadically and only in 5–10% is due to inherited mutations [25]. The risk of CRC development increases significantly in people with inflammatory bowel diseases (IBDs), such as ulcerative colitis (UC) and Crohn's disease (CD). Chronic inflammation processes induce development of colitis-associated cancers (CAC) generally initiated by mutations in *TP53* or by COX2 overexpression and followed by *APC* inactivation at later stages [26]. 

Although the genetic and epigenetic alterations responsible for the different gastrointestinal (GI) cancers are still unknown, a pivotal role of inflammation in their pathogenesis is emerged. In particular, COX2 overexpression contributes to this process inhibiting apoptosis and promoting angiogenesis and invasiveness of tumor cells. Concordantly, epidemiologic studies have demonstrated that the long-term and regular use of nonsteroidal anti-inflammatory drugs (NSAIDs, COX2 inhibitors) reduces the mortality from digestive tract malignancies [24, 25]. 

**Figure 2:**
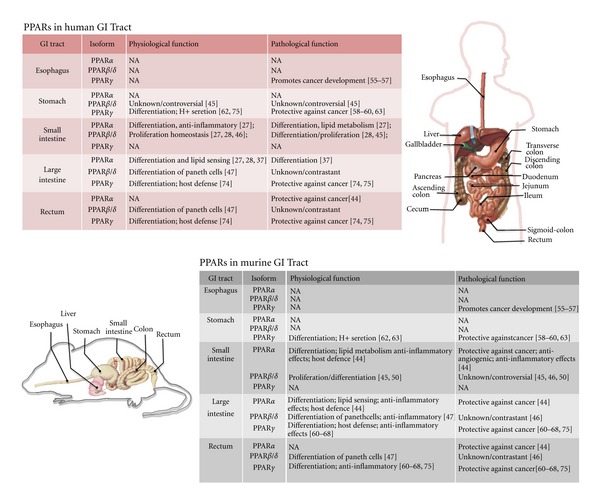
Physiological and pathological functions of the three PPAR isoforms in the human and murine GI tract.

In search of new strategies for the treatment of GI cancers, PPARs have attracted increasing attention mainly because of their anti-inflammatory effects, accompanied by prodifferentiation and proapoptotic functions [23–26]. PPARs are heterogeneously expressed along in GI tract and their role in the pathophysiology of these neoplasms is beginning to emerge (Figure 2) [27–29].

## 3. PPAR***α***

PPAR*α* is mainly expressed in the mucosa of the small and large intestine where “senses” the total flux of dietary fatty acids delivered [27, 28]. In these contexts, PPAR*α* regulates genes involved in lipid metabolism, inflammation, cell cycle progression, and angiogenesis [30–34]. Given its role in these latter processes, PPAR*α* has been suggested to contribute to tumor formation and/or progression. To date, no data are available on its involvement in GC and EC, whereas its role in CRC has been investigated both *in vitro* and *in vivo*. PPAR*α* is correlated with a reduced expression of MYC-related genes, such as cyclin D1, caspase3, NF*κ*B, STAT1, and EGFR. PPAR*α* activation inhibits capillary tube formation *in vitro *and angiogenesis *in vivo* through a mechanism that involves deconstruction of the cytoskeleton, reduction of bFGF-induced Akt activity and COX-2 expression. PPAR*α* also reduces the neovascularization, modulating the expression of VEGF, FGFs, members of the arachidonic acid P450 mono-oxygenases, thrombospondin, and endostatin [31–35]. *In vitro* PPAR*α* induces apoptosis through modulation of Bcl-2 and Bad proteins [35, 36]. PPAR*α* ligands, in addition, downregulate oncogenes and upregulate antiproliferative genes, supporting a tumor suppressor role [37]. In CRC cell lines, PPAR*α* is modulated by the activation of the MAPK pathways; specifically, phosphorylation of specific amino acid residues located at the PPAR*α* N-terminus region by JNK and p38 MAPK enhances its ligand-dependent transcriptional activity. This, in turn, promotes apoptosis, differentiation, and anti-inflammatory effects mainly through inhibition of iNOS, COX-2, and TNF-*α*. On the contrary, activation of ERK-MAPK signaling pathway reduces PPAR*α* activity [38, 39]. Growing evidence obtained in animal models suggests that PPAR*α* has anti-inflammatory effects *in vivo* but the precise and direct role it plays in intestinal inflammation is not fully elucidated. The data indicate that PPAR*α* has anti-inflammatory effects in a mouse model of chemically induced colitis; PPAR*α*-deficient mice exhibit enhanced inflammation; exposure to PPAR*α* ligands controls colonic inflammation through inhibition of proinflammatory cytokines. Collectively, the evidence supports that PPAR*α* activation leads to mitigation of IBD progression [40–42]. Unfortunately, the precise and correct assessment of PPAR*α* function in CRC is made even more complicated by species-specific differences. The data obtained from mice models indicate that PPAR*α* ligands play a potential role in suppressing polyp formation in *Apc*-deficient mice, an animal model corresponding to human familial adenomatous polyposis [43]. A significant reduction in PPAR*α* expression is detected in human CRC specimens and UC patients' mucosa, suggesting PPAR*α* as a therapeutic target to prevent adenoma formation also in IBD-induced cancer formation [36, 41, 44]. Thus, in CRC PPAR*α* seems to act as a tumor suppressor with antiangiogenic, anti-inflammatory, and, ultimately, antitumor activities. 

## 4. PPAR***β***/**δ**

To date, no studies have demonstrated a role for PPAR*β*/*δ* in the esophageal epithelium. In gastric epithelium, it is highly expressed but whether it has any role in tumorigenesis is still poorly understood [27, 28]. *In vitro* studies report an inverse relationship between PPAR*β*/*δ* and NF*κ*B, IL-1*β*, COX2, and the Wnt-*β*-catenin/TCF-4 pathways, suggesting a possible protective role in cancer development by virtue of its anti-inflammatory effects [45]. 

PPAR*β*/*δ* is also involved in the homeostatic regulation of proliferation/differentiation and modulation of the inflammatory response in cells of the small and large intestine [27, 28, 46]. Its physiologic role, however, is still unknown as well as it is controversial its function in inflammation and CRC development. In mouse models, PPAR*β*/*δ* activation by selective ligands in small and large intestine induces terminal differentiation of epithelial and Paneth cells that play an important role in immunity and host defense [27, 28, 46–48]. Emerging evidence suggests also that PPAR*β*/*δ* can suppress inflammatory bowel diseases through a PPAR*β*/*δ*-dependent and ligand-independent downregulation of inflammatory signaling [47, 48]. These effects may be due, in part, to interference with NF*κ*B signaling or, alternatively, to inhibition of Paneth cell differentiation that, in turn, could contribute to exacerbate experimentally induced colitis in PPAR*β*/*δ*-null mice [47, 48]. In contrast, administration of a highly specific PPAR*β*/*δ* ligand does not ameliorate inflammation [49]. The role that PPAR*β*/*δ* serves in the interplay between inflammation and cancer and in colon carcinogenesis remains debatable. In fact, *in vivo* and *in vitro* experiments have provided conflicting results suggesting that PPAR*β*/*δ* ligand activation can either potentiate or attenuate the process [50]. Its expression and/or activity is increased after loss of *APC* or activation of *K-RAS* expression [51, 52]. PPAR*β*/*δ* has also been shown to be a target of APC/*β*-catenin/T-cell factor- (TCF-) 4-pathway and, in turn, to modulate further downstream targets, such as c-myc and cyclin D1 [53]. 

## 5. PPAR**γ**

PPAR*γ* is the best-studied isoform in the GI cancer context. Its role in esophageal cancer development is debated: its activation *in vitro* reduces cell growth and induces apoptosis, implying that PPAR*γ* ligands could have a potential use as chemotherapeutic agents in the treatment of patients affected by dysplastic Barrett's esophagus [54]. In contrast, xenografted mice treated with PPAR*γ* agonists show an increased tumor growth. This discrepancy has been ascribed to *in vivo* effects of “tumor interactions,” differences in PPAR*γ* activation magnitude and PPAR*γ*-independent effects of thiazolidinediones. Recently, it has been reported that PPAR*γ* expression increases in less differentiated human Barrett's adenocarcinoma, supporting a role for PPAR in inhibiting the development of these tumors [54–57].

As far as GC, PPAR*γ* agonists reduce the proliferation of human cells lines *in vitro* although the effects appear to be dependent upon cell differentiation [58–60]. In contrast, PPAR*γ* silencing in GC cell lines reduces cell viability, suggesting that PPAR*γ* overexpression may induce tumorigenesis [61]. PPAR*γ* agonists induce gastric acid secretion via serum and glucocorticoid inducible kinase (SGK1) upregulation [62]. Although this stimulation should favor the formation of gastric ulcers, PPAR*γ* agonists have been reported to foster ulcer healing, suggesting that the potentially “dangerous” effect on gastric acid secretion is overridden by the simultaneous protective effects [62]. The critical importance of PPAR*γ* in gastric carcinogenesis *in vivo* has recently been provided: PPAR*γ* heterozygous-deficient mice show an increased susceptibility to carcinogen-induced GC and shorter survival rate than PPAR*γ* wild-type bearing mice, implying a tumor suppressor function. In this animal model, thiazolidinediones act as chemopreventive agents in a PPAR*γ*-dependent manner [63]. 

Several studies have addressed the role of *PPARG* in CRC development. *PPARG* mRNA is detected in the normal human mucosa of the caecum and colon, as well as in adenocarcinomas and CRC-derived cell lines. Although PPAR*γ* function in colon carcinogenesis has been controversial for long time, more recent evidence supports a role as tumor suppressor [64–68]. PPAR*γ* agonists induce cell cycle arrest, differentiation, and apoptosis. In particular, p16, p21 and p27, as well as the tumor suppressor gene, PTEN are upregulated while *β*-catenin, COX-2, VEGF, Bcl-2, and NF*κ*B target genes are downregulated. Finally, PPAR*γ* reduces the epithelial mesenchymal transition (EMT), a well-known process that allows cancer cells to acquire invasive ability, a prerequisite for metastasis formation. Consistent with the evidence *in vitro*, mouse models have shown that PPAR*γ* ligands reduce the growth of tumors originated from subcutaneously injected human CRC cells and the number of aberrant crypt foci (ACF) in a chemically induced model of IBD [60, 69]. 

Loss-of-function mutations in *PPARG* have been reported in 8% of human CRCs, implying a protective effect [70]. Although these mutations have been classified as “very rare events” [70, 71], increasing evidence suggests that PPAR*γ* activity is attenuated during the transition from adenoma to adenocarcinoma, likely explaining why PPAR*γ* agonists are effective in blocking the early stages of tumorigenesis (i.e., ACF formation is inhibited while little or no effect is detected in advanced tumor stages) [71–74]. PPAR*γ*-reduced activity may, at least in part, involve its phosphorylation by the mitogen-activated kinase ERK1/2, and its ligand-independent SUMOylation, two posttranslational modifications that negatively modulate PPAR*γ* activity [72, 73]. In addition to loss-of-function mutations and inactivating posttranslational modifications, low *PPARG* expression has been found in 35% of sporadic CRCs due to epigenetic events such as DNA methylation and repressive histone modifications [75–77]. Interestingly, the epigenetic repression appears to be associated with a more aggressive course, EMT activation, and patients' worse prognosis, further supporting the notion that PPAR*γ* is an independent prognostic factor in CRC [75, 78]. Reduced PPAR*γ* levels have been detected in patients affected by IBDs, such as UC and CD, suggesting that impaired* PPARG *expression precedes and is not secondary to the inflammatory process and likely contributes to the pathogenesis of IBDs [79–81]. Concordantly, *PPARG* genetic variations have recently been correlated with a different risk of IBD incidence [81, 82]. Low PPAR*γ* levels have also been found in peripheral mononuclear cells of IBD patients in the absence of specific *PPARG* mutations. Epigenetic events or abnormal signaling pathways carried out by natural ligands or microorganisms of the colon microenvironment might account for the impaired *PPARG *expression in UC and CD patients [82].

## 6. PPARs and ncRNA 

PPARs deregulation during tumorigenesis of the GI tract has been attributed to gene mutations, altered levels of expression and, more recently, epigenetic modifications. These latter events, however, have been identified as “critical” only for *PPARG* expression while no evidence has been provided for their involvement in PPAR*α* and PPAR*β*/*δ* regulation [69, 76, 77]. A novel mechanism of gene regulation is emerging that involves noncoding RNAs (ncRNAs). They are recognized as important regulators of physiological and pathological processes playing critical roles in DNA structure, RNA production, protein translation, and protein functions [83]. The term ncRNA includes both micro-RNAs (miRNA) and long noncoding RNAs (lncRNA). MicroRNAs are small noncoding RNAs that inhibit protein translation or induce degradation of their target mRNAs upon binding to cognate recognition sites usually located in the 3′ untranslated region [84]. Over one third of protein-coding genes is potentially regulated by miRNAs thus affecting important biological functions among which tumorigenesis [84, 85]. A handful of miRNAs have been identified to promote or inhibit tumor initiation, progression, and metastasis, influencing oncogenes or tumour suppressor genes or acting as oncogenes or tumor suppressors themselves [86, 87]. Although the role of ncRNAs in the regulation of PPARs expression or activity is beyond the scope of this review, we would like to mention the miRNAs directly involved in PPARs regulation. miR-21 and miR-10b downregulate PPAR*α* in liver, while miR-506 targets this receptor in human CRC cell lines [88–91]. PPAR*γ* is negatively regulated by miR-27 and miR-130 family members in preadipocytes, hampering adipocyte differentiation [92–97]. In addition, miR-27 reduces PPAR*γ* expression in LPS-stimulated macrophages, inhibiting its anti-inflammatory activity [92]. More recently, miR-27 has been implicated in downregulation of PPAR*γ* in cardiomyocytes and also in neuroblastoma and breast cancer [95, 97]. miR-122 targets PPAR*β*/*δ* in liver [98]. Lastly, PPAR activity may also be repressed via miR-dependent targeting of PPARs-coregulators [99]. All together these observations indicate that miRNAs may exert coordinating and redundancy-limiting actions on the gene-expression networks controlled by PPARs [99].

In addition to miRNAs, also long noncoding RNAs (lcnRNAs) regulate nuclear receptors and thus, potentially, PPARs expression and activity. LncRNAs are, in general, defined as non-protein coding transcripts longer than 200 nucleotides that might directly affect gene expression through the interaction with transcriptional activators/repressors inducing or repressing gene transcription [83]. Several studies suggest a critical role of lncRNAs also in the epigenetic-dependent gene regulation by orienting chromatin-modifying factors/complexes to specific locations in the genome and in the nucleus [83, 100, 101]. The direct involvement of lncRNA in PPARs expression has not been described so far; the ncRNA SRA (steroid receptor RNA activator) has recently been reported to associate with PPAR*γ* and modulate transcription of PPAR-target genes [102]. A more recent work provides evidence of a new mechanism of nuclear receptor activity regulation: the ncRNA Gas5 acts as a decoy RNA inhibiting the activity of the glucocorticoid receptor on its target genes [103].

Although the relationship between PPARs and ncRNAs in cancer is only at the beginning to emerge, it is conceivable that miRNAs may regulate PPARs expression influencing the development of GI cancers at different levels.

## 7. Conclusions and Future Directions

Dietary, environmental, and genetic factors contribute to the etiology, pathogenesis and risk with of gastrointestinal cancers. The link between PPARs and environmental factors in the development of GI tumors is strong, reciprocal but still poorly understood at molecular level. Inflammation plays a crucial role in the development of premalignant lesions of the esophagus, stomach, and colon rectum that, up to now, has only indirectly been proved through expression/correlation studies. PPARs impact diverse aspects of cancer development such as signaling pathways, metabolic interactions, cell cycle, and inflammation. 

Here, we have overviewed the most recent evidence of the literature supporting the hypothesis that the events underlying chronic inflammatory conditions and their evolution towards GI tumors could be at least in part orchestrated by the pro- and antitumor effects mediated by PPARs. Their expression and activity in tumor cells are modulated by genetic and epigenetic alterations; miRNAs are emerging as a new pathogenetic player. Intriguingly, dietary and life styles as well as environmental factors may influence PPARs function and impact cancer predisposition through epigenetic mechanisms. Hence, understanding how the individual genetic background and environmental factors contribute to PPARs deregulation and hence to the establishment of an inflammatory status or a tumor condition is mandatory. The studies reported here suggest, in addition, a rationale for novel strategies in cancer treatment whereby PPARs ligands might directly interfere with tumor growth and promote anticancer activity. More direct data and deeper evidence are still awaited to appraise the benefits that these agonists may provide in the prevention and treatment of GI tract inflammations and tumors. Clinical trials suggest that PPARs ligands may not be so effective as a single agent in advanced tumors but they could be effective in combination with a classical chemotherapy and additional anticancer agents such as epigenetic drugs, recently introduced into the therapeutic armamentarium. Another promising translational outcome of these studies is the possibility to identify PPAR alterations in premalignant lesions so that they can be used as prognostic biomarkers. In conclusion, elucidation of these pathways could provide biomarkers or new therapeutic targets with broad implications for cancer prevention, risk prediction, and prognosis.
